# Supplementary material for: Comprehensive Analysis of Autophagy-Related Genes in Rice Immunity against Magnaporthe oryzae
Source: Plants (Basel). 2024 Mar 22;13(7):927. doi: 10.3390/plants13070927 (PMC11013097; doi:10.3390/plants13070927)
Supplement: Supplementary file 1 [file plants-13-00927-s001.zip › Supplementary Finure.pdf]

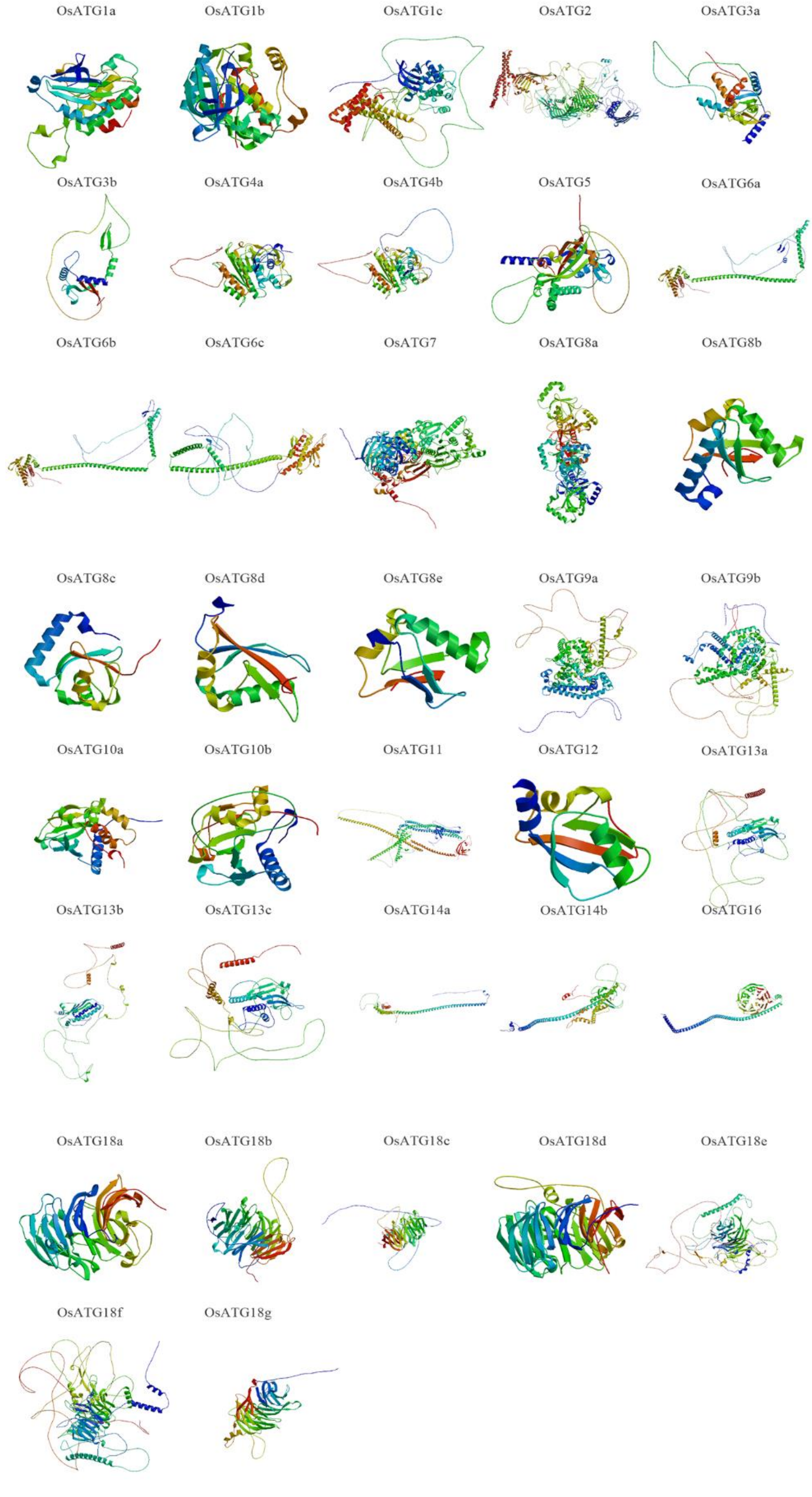

Figure S1. Tertiary structure analysis of OsATGs. The SWISS-MODEL website was employed to predict the 3D structure of the proteins encoded by OsATGs.

(a)

Anchor sequence: TAAAGCTTATGAGGCCTGTC
Degenerate sequence: GTCTGGAGCAGGAAA (Homozygous mutant)

Allele1: TAAAGCTTATGAGGCCTGTCGTCTGGAGCAGGAAA
Allele2: TAAAGCTTATGAGGCCTGTCGTCTGGAGCAGGAAA

Alignments of Allele1, Allele2, and reference sequence:
```
Allele1:   TAAAGCTTATGAGGCCTGTC------GTCTGGAGCAGGAAA (deletion)
Allele2:   TAAAGCTTATGAGGCCTGTC------GTCTGGAGCAGGAAA (deletion)
Reference: TAAAGCTTATGAGGCCTGTCTTCAACGTCTGGAGCAGGAAA
```

(b)

Anchor sequence: CTCAGGTTGAGCAGCCAATG
Degenerate sequence: GGGCTGTATGAGGTT (Homozygous mutant)

Allele1: CTCAGGTTGAGCAGCCAATGGGGCTGTATGAGGTT
Allele2: CTCAGGTTGAGCAGCCAATGGGGCTGTATGAGGTT

Alignments of Allele1, Allele2, and reference sequence:
```
Allele1:   CTCAGGTTGAGCAGCCAATG-----GGGCTGTATGAGGTT (deletion)
Allele2:   CTCAGGTTGAGCAGCCAATG-----GGGCTGTATGAGGTT (deletion)
Reference: CTCAGGTTGAGCAGCCAATGTGTCTGGGCTGTATGAGGTT
```

(c)

Anchor sequence: CAGGTTGGAGATGGCCAGGA
Degenerate sequence: ACTTCCTTCAAGCTC (Homozygous mutant)

Allele1: CAGGTTGGAGATGGCCAGGAACTTCCTTCAAGCTC
Allele2: CAGGTTGGAGATGGCCAGGAACTTCCTTCAAGCTC

Alignments of Allele1, Allele2, and reference sequence:
```
Allele1:   CAGGTTGGAGATGGCCAGGAACTTCCTTCAAGCTCG (insertion)
Allele2:   CAGGTTGGAGATGGCCAGGAACTTCCTTCAAGCTCG (insertion)
Reference: CAGGTTGGAGATGGCCAGGA-CTTCCTTCAAGCTCG
```

(d)

Anchor sequence: CTTTGTGGCAAGCACCAGAG
Degenerate sequence: TGTCACAGGGATGAT (Homozygous mutant)

Allele1: CTTTGTGGCAAGCACCAGAGTGTCACAGGGATGAT
Allele2: CTTTGTGGCAAGCACCAGAGTGTCACAGGGATGAT

Alignments of Allele1, Allele2, and reference sequence:
```
Allele1:   CTTTGTGGCAAGCACCAGAGTGTCACAGGGATGATA (insertion)
Allele2:   CTTTGTGGCAAGCACCAGAGTGTCACAGGGATGATA (insertion)
Reference: CTTTGTGGCAAGCACCAGAG-GTCACAGGGATGATA
```

(e)

Anchor sequence: TGTGGAGTGGAAGCGCTCGA
Degenerate sequence: TGTCAGGGGCAAAAC (Homozygous mutant)

Allele1: TGTGGAGTGGAAGCGCTCGATGTCAGGGGCAAAAC
Allele2: TGTGGAGTGGAAGCGCTCGATGTCAGGGGCAAAAC

Alignments of Allele1, Allele2, and reference sequence:
```
Allele1:   TGTGGAGTGGAAGCGCTCGATGTCAGGGGCAAAACC (insertion)
Allele2:   TGTGGAGTGGAAGCGCTCGATGTCAGGGGCAAAACC (insertion)
Reference: TGTGGAGTGGAAGCGCTCGA-GTCAGGGGCAAAACC
```

(f)

Anchor sequence: CGGGGAGGGGCTCGCCGTGA
Degenerate sequence: GCGGTGCATCACGCT (Homozygous mutant)

Allele1: CGGGGAGGGGCTCGCCGTGAGCGGTGCATCACGCT
Allele2: CGGGGAGGGGCTCGCCGTGAGCGGTGCATCACGCT

Alignments of Allele1, Allele2, and reference sequence:
```
Allele1:   CGGGGAGGGGCTCGCCGTGA-------------GCGGTGCATCACGCT (deletion)
Allele2:   CGGGGAGGGGCTCGCCGTGA-------------GCGGTGCATCACGCT (deletion)
Reference: CGGGGAGGGGCTCGCCGTGAACCGGGCGTACAAGCGGTGCATCACGCT
```

(g)

Anchor sequence: GGGCTCGCCGTGAACCGGGC
Degenerate sequence: AAGCGGTGCATCACG (Homozygous mutant)

Allele1: GGGCTCGCCGTGAACCGGGCAAGCGGTGCATCACG
Allele2: GGGCTCGCCGTGAACCGGGCAAGCGGTGCATCACG

Alignments of Allele1, Allele2, and reference sequence:
```
Allele1:   GGGCTCGCCGTGAACCGGGC----AAGCGGTGCATCACG (deletion)
Allele2:   GGGCTCGCCGTGAACCGGGC----AAGCGGTGCATCACG (deletion)
Reference: GGGCTCGCCGTGAACCGGGCGTACAAGCGGTGCATCACG
```

Figure S2. Verification of mutants. (**a**) Verification of *osatg6b*. (**b**) Verification of *osatg6c*. (**c**) Verification of *osatg8a*. (**d**) Verification of *osatg9a*. (**e**) Verification of *osatg9b*. (**f**) and (**g**) Verification of *osatg13a*. All mutants were generously provided by Prof. Qingjun Xie (South China Agricultural University, Guangzhou, China).
